# Medical education in Nigeria and migration: a mixed-methods study of how the perception of quality influences migration decision making

**DOI:** 10.15694/mep.2020.000001.2

**Published:** 2020-10-12

**Authors:** Eddy Awire, Mamodesan T Okumagba

**Affiliations:** 1 Delta State University

**Keywords:** Medical education, medical students, perception of quality, migration aspirations, migration, migration history.

## Abstract

This article was migrated. The article was marked as recommended.

Medical education in Nigeria faces numerous challenges and problems; a lack of a coherent admission policy, inadequate funding, poor planning, and erosion of values have led to a general perception of low standards and quality. In the face of these, questions arise as to their influence on medical students’ aspirations and intentions to migrate after graduation. This study uses a sequential mixed-method design to examine the extent to which the perceptions of the quality of medical education in Nigeria affect the aspirations and plans of Nigerian medical students to migrate after graduation. 211 final year students (out of a potential 580) participated in a survey; aged between 20 and 45. While the survey showed that the students perceived medical education to be of sufficiently good quality across a spectrum of variables, interview respondents described a dysfunctional medical education that failed to meet their aspirations. The perception of a declining standard in medical training is a major issue for Nigerian medical students and graduates. The inability to halt the decline in the quality of medical training in Nigeria, therefore, leaves many medical students and graduates feeling inadequately trained, and inadvertently feeds their desires and aspirations to migrate abroad after graduation.

## Introduction

The Nigerian education system is beset by numerous challenges and problems: a lack of coherent admission policy, resulting in a corrupted admission process ( Fatunde, 2009), inadequate funding, poor planning, and erosion of values have led to serious problems in the education sector. Moja (2000) observed that Nigerian institutions operate at a higher capacity than they were originally designed for and argued that this has led to a general perception of low standards and quality of education and that this leads to conditions that encourage brain-drain. Other studies on the education system in Nigeria reached similar conclusions ( FGN, 2012; Anyebe, 2014; Halidu, 2016). The new economics of labour migration (NELM) theory points to failures in the system, like the structural dysfunction in the Nigerian medical education system, as a reason individuals and families engage in international migration; they want to insure their members against such failures ( Massey *et al.*, 1993; Hagen-Zanker, 2008). In the face of these challenges, questions arise as to the influence of the structural failures in the education system on medical students’ aspirations and intentions to migrate after graduation. Medical training institutions in Nigeria are critically important to the effective functioning of the healthcare system and the health of the population: currently, they produce between 2000 and 3500 medical doctors each year ( Labiran *et al*., 2008; Omoluabi, 2014; Daily Trust, 2015). A fault in the production line of these important institutions, especially that which affects the quality of the products - the graduates - will also affect the quality of healthcare service delivery and the health of the Nigerian people. The failures of the Nigerian healthcare system can, therefore, be partly blamed on the dysfunction in the medical education and training system.

This study examines the extent to which the perceptions of the quality of medical education in Nigeria affect the behaviours, aspirations, and plans of Nigerian medical students and graduates to migrate abroad following graduation.

List of acronyms and abbreviations

UI University of Ibadan

UNIBEN University of Benin

UNIJOS University of Jos

UNN University of Nigeria, Nsukka

## Methods

### Study design, setting, and sampling

A two-phased mixed methods study that utilized a cross-sectional survey of final year medical students in four medical schools ( *Table 1*) and semi-structured interviews with final year medical students, some parents of final year medical students, house officers, resident doctors, faculty staff including clinical consultants, lecturers, and senior administrative staff of two medical schools in Nigeria ( *Table 1*).In the first phase (surveys), four medical schools were selected by probability sampling; a sample frame was drawn from the Medical and Dental Council of Nigeria (MDCN) list of all accredited schools, from which a cluster random sample was drawn. To ensure fair representation (of northern and southern medical schools) we divided the schools into six groups along the geopolitical zones (regions) of Nigeria and selected one medical school from each. The final year class in each selected school was purposively chosen to for the survey because they (final year class) were best suited to answer the questions in our questionnaire. However, for security and safety reasons (The Federal Government of Nigeria was, and still is, waging a ‘war on terror’ against the Islamic sect ‘Boko Haram’ in the North East and North-West of Nigeria, and at the time of our field work a ‘state of emergency’ (Martial Law) was in place in the North-East zone) the surveys were not carried out in two regions (North-East and North-West).

In the second phase (interviews), two schools (and interview participants) were purposively selected based on their migration history and for scoring high for students’ aspirations to migrate from the earlier survey (phase 1) of the four medical schools above.

**Table 1.** Study participants

SURVEY			
Region	Medical school surveyed	Admission quota	Participants
South-West	University of Ibadan, Ibadan	180	52
South-East	University of Nigeria, Nsukka	150	50
South-South	University of Benin, Benin City	100	54
North-Central	University of Jos, Jos	150	55
**INTERVIEWS**
**Participants**		**University of** **Ibadan**	**University of** **Benin**
Students		6	4
House officers ^1^		2	2
Staff (academic & non-academic staff)		5	5
Family members (parents) ^2^		1	2
**Total**		**14**	**13**

_^1^_Our study was designed as an embedded multiple case study of two (2) medical schools, with the medical schools (cases) as primary units of analysis. The embedded units of analysis were two students (and their families), one from each of the schools (the cases), each with a positive history of family migration, and purposively selected to explore the role of the family in migration decision making. As a result, only two families were involved in the study; and only three of the four parents were available for interviews for the study. These details and findings, however, are presented in another report that is under consideration for publication.

_^2^_The transition from final year studentship to a House officer, and from a House officer to a resident doctor is very fluid. In the duration of our study many of the students became House officers.

Ethical approval for this study was granted by the National Health Research Ethics Committee of Nigeria (NHREC). However, in each of the institutions where this study was carried out, ethical clearance was obtained from their own ethics review committees.

### Participant recruitment and data collection

The initial contact with each of the medical schools was made by contacting the office of the school secretary. Following introductions to staff and students, the possibility of their participation in the study was discussed; those who indicated interest in the study were left with a letter of introduction explaining the reasons for the study. For the surveys, dates were then established after discussions with both the course advisers and the class representatives for the final year classes. On the day(s) of the survey(s) (and interviews) in each school, a consent form was signed by each potential participant before being recruited for the study. For the interviews, each potential participant approached was asked for suggestions as to who they believed would be helpful in exploring and discussing the topic *.*

The questionnaire for the survey was piloted in a medical school not included in this study. Following the preliminary analysis of the pilot survey data, the pilot questionnaire was amended to arrive at the final survey questionnaire ( *Supplementary file 1*). The final year class (in each of the schools) was purposively chosen for the survey because they were best suited to answer the questions in the questionnaire.

The interviews were conducted following a multiple-topic interview guide that served both as a prompt and a reminder of the information that needed to be collected. Interviews lasted between 30 and 45 minutes, were conducted in the offices, homes or hostels of participants, and were digitally recorded with the consent of participants.

### Data management and analysis

The data from the survey were entered into IBM SPSS statistics (version 21), summarised, and presented with appropriate descriptive statistics (means, frequencies, percentages, standard deviations, etc.), charts and cross-tabulations. The relationships between variables were statistically explored, looking at correlates of positive attitudes to migration at a number of levels.

Following completion of the fieldwork, audio files of the interviews were transcribed word-for-word into Microsoft Word. Interview transcripts were transferred to NVivo 10 for organization and management, sorted into themes, categories, and codes, leading to the eventual coding of the study’s interview data.

## Results/Analysis

### Perception of the quality of medical education

211 final year students (out of a potential 580) participated in the survey, aged between 20 and 45, with a median age of 24 (interquartile range 23 to 25). They were mostly males (61.6%), single (97.2%) and Christian (93.8%), with 78.2% coming from an urban background. 63.5% of respondents viewed migration positively while close to half (41.7%) aspired to migrate after graduation ( *see Appendix 1 for Summary of key survey findings, and Appendix 2 for a summary by institutions*).

While most of the survey respondents perceived their medical education to be of sufficiently good quality across a spectrum of variables ( *Table 2*), the views expressed by interview respondents were different: from academic staff complaining of difficulty in coping with the increasing student population, to students claiming that their medical education was “stereotyped towards the theoretical aspect” rather than the clinical aspect of learning, and parents of the medical students believing that the medical education their children were receiving was not good enough to prepare them for practice; the interview respondents perceived medical education in Nigeria to be deficient.

**Table 2.** Perception of the quality of medical education in Nigeria

Variable	Excellent N (%)	Very Good N (%)	Good N (%)	Indifferent N (%)	Bad N (%)
Experience studying medicine	26 (12.3)	70 (33.2)	88 (41.7)	23 (10.9)	4 (1.9)
Quality of instruction	5 (2.4)	34 (16.0)	113 (53.6)	28 (13.3)	31 (14.7)
Quality of training facilities ^x^	4 (1.9)	19 (9.0)	111 (52.6)	36 (17.1)	40 (19.0)
Quality of support	9 (4.3)	28 (13.3)	98 (46.4)	44 (20.9)	32 (15.2)
Relevance of curriculum	12 (5.7)	60 (28.4)	105 (49.8)	26 (12.3)	8 (3.8)

^X^ Missing response - 1 (0.5%)

### Quality of instruction

They way students felt about the quality of the instruction they were receiving from teaching staff affected their perception of the quality of education they were getting in medical school. Although most of the students surveyed (72.0%) in this study felt happy with the quality of instruction, over a quarter (28.0%) were critical of the quality of instruction they were receiving, describing it as inadequate ( *Table 2*). Some believed that the system forced them to strive principally to pass exams, rather than acquiring the knowledge needed for professional practice:

*“... it’s a joy for most lecturers here that students do not pass, though they deny it. And it breeds the culture of doing everything just to pass, not learning everything you need to learn.”* (24-year old female student).

With their minds set on getting it right, students focus their attention on the things that have been shown to help in passing exams, like “... *studying past examination questions and how to answer them, rather than studying with textbooks*” (25-year old male student). They (students) also described their assessment through oral examinations as being too subjective. Some lamented that when it comes to their assessments, they were at the mercy of their examiners:

*“if they don’t like you, you are done.*” (26-year old male student).

They described their assessment process to be so heavily weighted, by the academic staff, in favour of the oral examinations, that they had to do everything they could to remain in the staff’s good books, and do, strictly, only what they have been instructed to do because “... *the goal here is to pass”* (29-year old female student) *..* They believed that this limited them within the scope and boundaries of their lecturers, some of whom they claimed made little or noefforts to bring their teaching methods up to speed and in line with advancements in modern-day teaching.

### Quality of medical training facilities

Most of the complaints by staff and students in this study were related to issues with training facilities, which they described as not being fit for purpose. Facilities were either unavailable *,* or the available ones were either non-functional or in a bad state *.* The unavailability of training facilities (or the poor state of those available) put a lot of pressure on the teaching and clinical staff, who were obliged to devise ways to deliver the medical curriculum with whatever facilities that were available (and functioning).

*“It’s either they (facilities) breakdown every time or after breaking down replacing them takes a long time.”* (Registrar).

The survey of students in this study revealed that the way they perceived the quality of their training facilities was significantly (p = 0.016) associated with the way they viewed the migration of doctors from Nigeria; of those who felt the quality of their academic training facilities was poor, almost two-thirds (62.5%) viewed migration positively; suggesting that perceiving the training facilities as poor leads to attitudes that favour migration. Over a third (36.1%) of students in this study expressed dissatisfaction with the state of their medical training facilities ( *Table 2*). One 24-year old male student described his pre-clinical training facilities and training to be as good as “... *having no education*”.

The lack of an equipped clinical skills acquisition facility for training students was a common complaint from both staff and students. It meant that students had to learn as they carried out procedures on patients at the hospital, consequently students *“... hardly have the chance to make errors and learn from their errors”* (26-year old male student) *.* The combination of a lack of skills-acquisition facilities and limited exposure to hands-on clinical training led directly to improvising in the absence of the necessary resources. One resident doctor stated: *“We have adapted to making use of what we have; we improvise a lot”.* Although improvising can be a necessary and sometimes life-saving measure, both staff and students agreed that good clinical standards were gradually, but increasingly, being lost to improvisation.

### Quality of support

The way students perceived the quality of support they received from their training institutions was significantly (p = 0.002) associated with the way they viewed the migration of doctors. A significantly higher proportion of those who viewed migration negatively perceived the quality of support received from their institutions as *very good* (33.3%) or *excellent* (16.7%), compared to those who viewed migration of doctors positively ( *very good*, 14.9%: *excellent*, 5.2%): suggesting that those who were happy with the support they received from their training institutions were much more likely to view doctors migrating from Nigeria negatively, while those who were not happy with the support were much more likely to view the migration of doctors positively. Over a third (36.1%) of students surveyed were unhappy with the support they were receiving ( *Table 2*).

Although students in this study acknowledged the existence of course advisers at every level of their medical school training, most of them also said they had never consulted their course advisers largely because they preferred getting advice from fellow students who were senior colleagues. Students described “... *being scared*” of approaching most of their professors and consultants *“... because there is too much hierarchy”* in the medical school. Such atmosphere led students to perceive the support they receive from their institutions in a less than positive way. In one of the medical schools, the management went as far as appointing named staff advisers to serve as ‘mentors’ for each student. However, students complained that they never heard back from the school nor their supposed mentors, with some citing this as one of the reasons they never sought counselling/advise from staff.

### Experience of studying medicine

In this study, respondents’ aspirations to migrate were significantly (P = 0.034) associated with the way they felt about their experience of studying medicine: almost two-thirds (60.0%) of those who expressed a strongly negative aspiration to migrate claimed they had an excellent experience studying medicine, whereas only a third (35.3%) of those who expressed a strongly positive aspiration to migrate felt the same. This suggests that those who felt positive about their experience of studying were less likely to aspire to migrate after graduation. Although most of the survey respondents (87.2) described their experience of studying medicine as positive ( *Table 2*), for those who claimed their experiences were not so great (12.8%), this was an issue.

*“It was the poor teaching, the poor facilities, and the poor experience that strengthened my resolve to want to do better with myself; to want to go abroad and further my medical training.”* (27-year old female student)

While almost equal percentages of students wished to pursue their specialty training abroad (33.2%) and in Nigeria (31.3%) ( *Table 3*), some of them also expressed serious concerns about the training programs in Nigeria. They pointed to the general socio-economic and political situation in Nigeria, their perception of the quality of medical education in Nigeria, and especially, the experiences of resident doctors in Nigeria, as reasons they would rather embark on their post-graduate training outside the country.

**Table 3.** Post-graduation career plans

Career plan post-graduation	N (%)
Full-time practice in Nigeria	19 (9.0)
Specialty training in Nigeria	66 (31.3)
Research/academics in Nigeria	1 (0.5)
Full-time practice abroad	11 (5.2)
Specialty training abroad	70 (33.2)
Research/academics abroad	8 (3.8)
Undecided	36 (17.0)
Total	211 (100%)

Students described what they had seen resident doctors experience in their post-graduate training as ‘ *uninspiring*’.

*“*[...] *when I saw the way resident doctors were being trained here in Nigeria, and I saw my future; it wasn’t something I was looking forward to,* [...] *at that point, going abroad was my only option.”* (28-year old male student).

Respondents complained of the poor rapport between consultants and resident doctors, describing an atmosphere of fear, with too much of a hierarchy. Students, house officers, registrars, and some senior registrars described how some consultants disrespected resident doctors during clinical ward rounds, in the presence of students, nurses, patients and sometimes, patients’ relatives.

*“It starts with the consultants talking down on the senior registrars; the senior registrars then talk down on registrars, who now talk down on house officers and students”.* (Senior registrar)

A resident doctor lamented how the *“... humiliating experience deals ones’ ego such a blow that for days you feel like quitting and not returning to training afterward”.* One 32-year old female student recalled how they (students) try to avoid engaging such residents in *“... eye-to-eye contact afterward”*, just so they don’t add to their misery, and *“... feeling pity for residents”* and *“... wishing never to be in their position ever”.* She described the fear of being *“... picked on”* for similar treatment, and how, to avoid being victims of similar treatment they (students) stayed quiet, afraid to ask questions. Students and house officers also pointed to the heavy workload of resident doctors as a factor that discourages them. They explained that to meet the demands of their training, residents spent a lot of their time in the hospital rendering a variety of services, leaving them with little time to prepare for exams, putting them at a disadvantage when the exams arrive. Seeing residents disrespected and then struggle to pass exam after exam because they spent most of their time rendering services in the hospital made medical students regard the place of the resident doctors as “... *unenviable*” and “... *uninspiring*”. That perception invariably led them to look elsewhere to pursue their aspirations for postgraduate training.

### Frequent industrial action

Medical education in Nigeria grapples with the very frequent industrial action taken by unions, in the universities and in the healthcare system ( Omoluabi, 2014; Oleribe *et al.*, 2016; and Adeloye *et al.*, 2017). The effects of these industrial action stoppages are worse for medical students who, under normal circumstances, spend a minimum of six years in training. Illustrating how these incessant strikes by lecturers and doctors affected medical education, a 25-year old female student lamented:

“For a course that should last for six years, I ended up spending eight years without failing an exam or repeating a class.”

Good clinical skills are lost due to the interruptions to continuous hands-on training and this puts additional pressure on clinical staff as it usually means repeating some clinical procedures, just so they could bring students up to the minimum training standard. The incessant industrial action also serves as a ‘push factor’ for migration and has led many parents who can afford it to send their children to study in other countries where the education systems are more stable.

*“I don’t think I want to be here; if internship that should be 12 uninterrupted months gets interrupted, and you start getting extensions. Not to talk of how long you spent in school.”* (House officer)

Students and young medical graduates who have experienced these strikes would want to avoid experiencing them again.

### The desire to be the best and to be relevant

Nigerian medical students and doctors aspired to be the best in their fields, as well as to be relevant in any sphere of life in which they find themselves. Consequently, they felt the need to develop and improve themselves beyond what was available to them through the system in Nigeria. This general perception of the poor state and quality of medical education, in general, was a driver of the aspiration of both students and young medical graduates to migrate to a developed country. Respondents stated that they felt their training was inadequate, and that to achieve a ‘proper’ training and be among the best, one needed to “ *... go out and develop oneself*”. One resident doctor reflected:

*“*[...] *you can’t really get everything you need in school here. Most of the time you might need to go abroad to study more to at least broaden your horizon.”*

While another respondent, a 25-year old male student stated, “... *what I aspire for, we don’t have here in Nigerian medical schools and hospitals”.*

This feeling of the inadequacy of medical training was made worse by some professors who selectively told of their wonderful experiences studying or practicing medicine abroad; this whetted the appetite of students for migration as they too wanted to obtain foreign qualifications.

*“certificates gotten from foreign universities are much more respected than our own here.”* (House officer).

Residents lamented how this preference for foreign certificates affected the recruitment of doctors in general, and into residency training programs at teaching hospitals in Nigeria. According to one resident “ *the preference for foreign certificates sends a signal to students and young medical graduates that their training and certificates are not only deficient, but that they will be left out in the scheme of things if all they have are local trainings and certificates.*”

## Discussion

Most of the students surveyed in this study felt happy with the quality of instruction (72.0%) and support (64.0%) they were receiving from their faculty/schools. Most (87.2%) also described their experience of studying medicine as positive ( Table 2); and interview respondents felt the medical education they had received in Nigeria prepared them for passing the qualifying examinations abroad and practicing successfully. However, the medical education system in Nigeria was described by some respondents in this study as broken, dysfunctional, and in need of structural reform; they believed that students were receiving training that prepared them inadequately for their professional future. As a result, both students and graduates felt the need to develop themselves further; they also believed that post-graduate medical education in Nigeria was not equipped to provide them with the training they needed. This perception of a broken and dysfunctional medical education system was a major driver of the aspiration of medical students and graduates to migrate abroad for further training or practice; migrating abroad should therefore be seen as one of their strategies to protect and insure themselves against the risks of the failures and dysfunctions in the Nigerian medical education training system.

Close to half (41.7%) of students surveyed in this study aspired to migrate after graduation ( *see Appendix 1 for Summary of key survey findings, and Appendix 2 for a summary by institutions*). While this is high, other studies in Sub-Saharan African have reported similar findings ( Silvestri *et al.* 2015). In Ghana for example, Eliason *et al.* (2014) reported nearly half (49%) of respondents in their study aspired to migrate after graduation; Kizito *et al.* (2015) reported 44.6% of respondents in their study in Uganda wanted to migrate after graduation; a similar pattern was also reported in Malawi ( Bailey *et al.* 2012; Mandeville *et al*. 2012; Sawatsky *et al.* 2014).

Medical education in Nigeria faces a huge infrastructural challenge: an assessment of the needs of Nigerian Public Universities returned a damning report on their infrastructures ( FGN, 2012). Medical education is the worst-hit area in the education system; the healthcare sector, which also supports the training of medical students, suffers the same, if not a worse fate ( Nwosu, 2000; Ezenolue, 2011; Welcome, 2011) as the education sector. More than any other factor, the infrastructural challenges were the factor most frequently cited by the respondents in this study who said they were preparing to leave Nigeria. They did not believe that the medical training facilities in Nigeria were good enough to provide the training they would need in the future.

The increasing demand for medical education in Nigeria has led to a proliferation of medical schools in the country. However, as reported by Oguntoye (2000), the rapid expansion has not been matched with a commensurate investment in infrastructural development, or staff recruitment and development. Medical schools recruit academic staff, not on the basis of academic qualifications or teaching experience, but “... purely on their possession of specialist qualification” ( Ibrahim, 2007: 5). Not only are these specialists few in Nigeria (the completion rate of those in specialist training programmes in Nigeria is as low as 30% ( Hagopian *et al.*, 2005), but most are already clinical specialists in hospitals and have little motivation to take teaching or academic training. Ibrahim (2007: 5) for example, stated: “it would not be an exaggeration to say that the medical teachers in Nigeria are vastly untrained in teaching methods”. Staffing challenges are made worse by an academic and medical brain-drain ( Tankwanchi, 2012; Tankwanchi, Ozden and Vermund, 2013; Merçay, Dumont and Lafortune, 2015) occasioned by the factors previously described.

At the heart of the problems facing medical education in Nigeria is the under-funding of the system. Almost all the issues raised by this study’s respondents about the current quality of Nigerian medical education have the common denominator of poor funding, resulting in poor remuneration of staff, and failures in maintaining facilities in good working order. Government’s budgetary allocation to the public education sector is so inadequate that almost all of it goes into recurrent overhead costs, leaving little for infrastructural development ( Halidu, 2015). Some ( Kalama *et al*., 2012)have even accused the Nigerian government of prioritizing the emoluments of government officials above investment in the education or health of Nigerians *.* These challenges are made worse by the poor management practices of medical training institutions with regard to scarce financial, material and human resources for health and education. A lot of the factors implicated in leading to low motivation, dissatisfaction and the many industrial actions ( Oleribe *et al*., 2016; Adeloye *et al*., 2017), could have been avoided by better leadership in the management of healthcare institutions. For example, the complaints by students about the “... *uninspiring*” experiences of resident doctors at the hands of consultants could be easily resolved by management taking a firm position against residents, students, patients, and human beings, in general, being disrespected.

Some researchers have recommended expanding the residency training programmes ( Hagopian *et al*., 2005; Tankwanchi, Ozden and Vermund, 2013)and the introduction of exit requirements ( Frehywot, 2010; Kollar & Buyx, 2013). Findings from this study show that such measures would only delay the migration of those already dissatisfied with the system if not accompanied by structural changes leading to improvements in the training programmes. As the new economics of labor migration theory explains, people welcome the opportunity to participate in important systems in their local environment ( Massey *et al*., 1993; Hagen-Zanker, 2008). However, if structural failures in the system persist, they will seek ways to insure themselves against the risks of the failures; in most cases, their main strategy for doing this is to migrate from Nigeria. The residency training programmes in Nigeria need a holistic reform that should include better training facilities, better treatment of doctors in terms of remuneration and respect, and an improvement in conditions that would ensure a better training completion rate.

## Conclusion

Medical education in Nigeria faces a lot of challenges. The perception of a declining standard in medical training is a major issue for Nigerian medical students and graduates, who dream of and aspire to be among the best in their profession. They (Nigerian medical students and graduates) read about the joys of high-quality training and practice in other parts of the world where medical education and practice enjoy the benefit of being taken seriously. The inability to halt the decline in the quality of medical training and practice in Nigeria, therefore, leaves many medical students and graduates feeling the inadequacies of their training and the need for further training outside Nigeria. So, a dysfunctional medical education system in Nigeria inadvertently feeds the desires and aspirations of medical students and graduates to migrate to countries where they believe they can achieve their dreams and professional aspirations after graduation.

## Take Home Messages

• Medical training institutions in Nigeria are critically important to the effective functioning of the healthcare system and the health of the population
• The perception of a declining standard in medical training is a major concern for Nigerian medical students and graduates
• The inability to halt the decline in the quality of medical training and practice in Nigeria, leaves many medical students and graduates feeling inadequately trained
• A dysfunctional medical education system in Nigeria inadvertently feeds the desires and aspirations of medical students and graduates to migrate out of Nigeria
• Medical education in Nigeria needs a holistic reform.

## Notes On Contributors

**Dr. Eddy Awire** is a lecturer at the College of Health Sciences, Delta State University, Nigeria. His research interest includes medical education, and health systems, particularly, human resources for health.

**Dr. Mamodesan T Okumagba** is a lecturer in the Department of Community Medicine Department of the College of Health Sciences, Delta State University, Delta State, Nigeria. His research interest includes improving access to oral healthcare, health promotion, and promoting the use of digital technology to improve healthcare.

## Figures and Tables

**Table:** 

**Gender**	Malen (%) 130 (61.6)	Femalesn (%) 81 (38.4)	X	X	X	Totaln (%) 211 (100)
**Marital status**	Singlen (%) 205 (97.2)	Marriedn (%) 6 (2.8)	X	X	X	211 (100)
**Religion**	Christianityn (%) 198 (93.8)	Islamn (%) 11 (5.2)	African traditionn (%) 1 (0.5)	Nonen (%)1 (0.5)	X	211 (100)
**Views on migration**	Strongly negative n (%) 3 (1.4)	Negativen (%) 9 (4.3)	Neutraln (%) 65 (30.8)	Positiven (%)116 (55.0)	Strongly positiven (%)18 (8.5)	211 (100)
**Aspirations to migrate**	Strongly negative n (%) 5 (2.4)	Negativen (%) 27 (12.8)	Neutral n (%) 92 (43.6)	Positiven (%)70 (33.2)	Strongly positiven (%)17 (8.0)	211 (100)
**Migration history**	Nonen (%) 65 (30.8)	Neighbourn (%) 33 (15.6)	Friend or colleaguen (%) 13 (6.2)	Extended familyn (%) 53 (25.1)	Nuclear familyn (%) 47 (22.3)	211 (100)
**Reasons for studying medicine**	Saving livesn (%)128 (60.7)	Better incomen (%) 11 (5.2)	Social Prestigen (%) 4 (1.9)	Family pressuren (%)12 (5.7)	Childhood dreamn (%)8 (3.8)	Othersn (%) 48 (22.7)

**Table:** 

	**All**	**UI**	**UNIBEN**	**UNIJOS**	**UNN**
Personal migration history	16.6% positive	25.0% positive	14.8% positive	12.7% positive	14.0% positive
Family migration history	74.4% positive	86.5% positive	63.0% positive	81.8% positive	66.0% positive
Family member practised abroad	41.7% positive	63.5% positive	31.5% positive	38.2% positive	34.0% positive
Views on migration	63.5% positive	73.1% positive	64.8% positive	72.7% positive	42.0% positive
Migration aspiration	41.2% positive	61.5% positive	37.0% positive	38.2% positive	28.0% positive
Post-graduation plan	42.2% abroad	53.8% abroad	38.9% abroad	45.5% abroad	30.0% abroad
