# Supplementary material for: Medical education in Nigeria and migration: a mixed-methods study of how the perception of quality influences migration decision making
Source: MedEdPublish (2016). 2020 Oct 12;9:1. Originally published 2020 Jan 7. [Version 2] doi: 10.15694/mep.2020.000001.2 (PMC10712584; doi:10.15694/mep.2020.000001.2)
Supplement: Peer review history

# Reviewer report: Reviewer response for version 2

**DOI:** 10.21956/mep.20258.r31436

**Published:** 2021-02-17

**Authors:** Richard Hays

**Affiliations:** 1 James Cook University

This review has been migrated. The reviewer awarded 4 stars out of 5 This article addresses a globally important issue - the potential medical 'brain drain' from less developed to more developed nations. The reasons are complex and perhaps each nation has a rather unique combination of factors. The authors are adding to the evidence base through a more detailed look at one nation. Nigeria has traditionally had a strong medical education system but still many leave for opportunities elsewhere. However, there some concerns that the authors may wish to address. The first is that this is study of a single cohort year across four institutions with a relatively small response rate: these limitations should be acknowledged. The second is that the authors focus on the quality of Nigerian medical education, stating that this is deteriorating. I have no knowledge that supports this assertion and do not think that this study provides evidence for (perhaps) a perception that the quality is falling. Perceptions can be powerful - exploring that perception further would be a useful further study - but reasons for leaving may be multifactorial, as elsewhere.

Reviewer Expertise:

NA

No decision status is available

# Reviewer report: Reviewer response for version 2

**DOI:** 10.21956/mep.20258.r31435

**Published:** 2020-10-19

**Authors:** Itoro Udo

**Affiliations:** 1 St. Joseph's Healthcare, London, Ontario

This review has been migrated. The reviewer awarded 5 stars out of 5 Authors have admirably made changes to the study sampling, setting and sampling that justifies the methods used. In a way not often witnessed in medical education, the impossibility of conducting the study in certain parts of Nigeria demonstrates how political upheavals may interfere with medical education research. Their purposive sampling also recognizes the important role of family in the decision to migrate, in Nigeria. In comparing migration intentions in their study with those from other developing countries, authors have shown that intentions to migrate may be widely held at the final stages of medical training, in Africa. Future research may elucidate whether there may be different factors that galvanize action. The positive experiences of students studying medicine and their satisfaction with the quality of instruction they receive, are the most overt, perceived positive aspects of medical training in NigeriaI find their perception of the relevance of their curriculum interesting. This may be contested by graduates in diaspora. However, survey of learners, including their thoughts about their curriculum is a valid study method in other countries. Bottom line, medical graduates do move, in order to improve themselves. The second version of this paper was refreshing to read and would certainly add to the body of evidence in the area of medical migration.

Reviewer Expertise:

NA

No decision status is available

# Reviewer report: Reviewer response for version 2

**DOI:** 10.21956/mep.20258.r31434

**Published:** 2020-10-13

**Authors:** Ken Masters

**Affiliations:** 1 Sultan Qaboos University

This review has been migrated. The reviewer awarded 5 stars out of 5 The authors have addressed the concerns raised by the reviewers of Version 1, and have produced a paper that gives valuable insight to the perception of medical education quality, and how it influences migration decision-making among medical students in Nigeria. It is always difficult to highlight shortcomings in one’s own system, and the authors are to be commended for tackling a difficult subject head-on. Rectifying the situation will not be easy, and will be a long struggle; this paper, however, lays bare the issues that will need to be addressed, and may be the beginning of the process.I look forward to a paper in five years’ time that gives an indication of steps taken along the road.

Reviewer Expertise:

NA

No decision status is available

# Reviewer report: Reviewer response for version 1

**DOI:** 10.21956/mep.19124.r27610

**Published:** 2020-04-14

**Authors:** P Ravi Shankar

**Affiliations:** 1 American International Medical University

This review has been migrated. The reviewer awarded 4 stars out of 5 This is an interesting study about medical education in Nigeria and migration. The authors have carried out a mixed methods study in four universities in Nigeria. They have described the Methodology and the Results in detail. How many medical schools are there in the country? I assume there will be schools in both the government and the private sector and I am assuming all four institutions involved in this study are government colleges. I was concerned on hearing about the system of assessment followed in Nigeria and the dominance of oral examinations. Is the examination pattern uniform throughout Nigeria? Are there any proposed changes in assessment? What is the duration of the undergraduate medical course in Nigeria and do students join after twelve years of schooling? How are the students selected? What is the annual tuition fee and other expenses? Are students predominantly from higher socioeconomic backgrounds? Some of these factors may also influence migration. The hierarchical nature of the program is also common in many other countries. Students may feel reluctant to approach faculty members personally. The Discussion section has concentrated primarily on deficiencies in infrastructure and can be expanded to also address other areas mentioned in the Results. The authors can compare their findings with other studies from Nigeria and other developing nations. The appendices are informative. This is an important study and the authors can address comments by the reviewers and submit a revised version. This manuscript is likely to be of broad interest to medical educators.

Reviewer Expertise:

NA

No decision status is available

# Reviewer report: Reviewer response for version 1

**DOI:** 10.21956/mep.19124.r27607

**Published:** 2020-03-25

**Authors:** Ken Masters

**Affiliations:** 1 Sultan Qaboos University

This review has been migrated. The reviewer awarded 3 stars out of 5 An interesting study of how the perception of quality influences migration decision-making among medical students in Nigeria.The paper begins with a frank account of the problems facing medical education in Nigeria. The results, unfortunately, show that these problems appear to be deeply rooted, with little hope of improvement, unless drastic action is taken. One has a depressing sense that there is a vicious cycle, and, as strong candidates feel unhappiness with the current system and so desire to leave, the loss of these doctors will further worsen the medical education environment.There are some shortcomings to the paper, but I see that the other reviewers have already noted them. As the authors are currently undertaking a second version of the paper, there is no reason for me to repeat those here; rather, I look forward to seeing Version 2 of the paper in which those issues are addressed.

Reviewer Expertise:

NA

No decision status is available

# Reviewer report: Reviewer response for version 1

**DOI:** 10.21956/mep.19124.r27609

**Published:** 2020-01-17

**Authors:** Bunmi Malau-Aduli

**Affiliations:** 1 James Cook University

This review has been migrated. The reviewer awarded 3 stars out of 5 This study investigates how perceptions of educational quality impact on migration decisions for further training among Nigerian medical students. The research topic is of high relevance and the authors adequately present the challenges faced by the educational sector in Nigeria. However, the various reasons for the migration/brain drain from the country or from the region (Africa) were not fully elucidated. Current evidence suggests that reasons for physician migration from low-income to high income countries range from economic and political instability, low remuneration, poor training and work environment. Also, what is the rationale for including parents in the study and why did the interview participants come from only the south? Given that there are distinct regional differences in the Nigerian educational system, it would have been interesting to see if there were divergent views between these participant groups.

Reviewer Expertise:

NA

No decision status is available

# Reviewer report: Reviewer response for version 1

**DOI:** 10.21956/mep.19124.r27608

**Published:** 2020-01-08

**Authors:** Itoro Udo

**Affiliations:** 1 St. Joseph's Healthcare, London, Ontario

This review has been migrated. The reviewer awarded 4 stars out of 5 The study by Awire and Okumagba was read with interest. The authors have given a background that justifies this study. They have drawn on literature and evidence in the area of tertiary education in Nigeria. However, what may have improved this section would have been additional literature and evidence of how the state of healthcare and facilities may be linked to intention to migrate. This is important because the training of doctors involves both academic and clinical aspects. I am not clear about how the medical schools were selected, given the number and spread of medical schools in Nigeria. I would suggest that some Nigerians would join issues with the consideration of UNIBEN as representing South-South geographical area. Though low in numbers, the inclusion of the views of parents, as stakeholders, improved the quality of this study. The results of the study are wide ranging and open up areas for further of research which may benefit from qualitative exploration. The discussion needed to be more balanced. The perceived negative aspects of training are highlighted but the positives are omitted thereby affecting the picture being painted. There may be aspects of training that enables migrating doctors to succeed in their host countries or health systems. As a primary medical Nigerian graduate, with interest in medical education, who had migrated, this is a study that I have been wanting to see for years. This is a good early effort from the authors, starting to elucidate the complexities of medical migration as regards to Nigeria, which is a major contributor to global medical migration.

Reviewer Expertise:

NA

No decision status is available

# Reviewer report: Reviewer response for version 1

**DOI:** 10.21956/mep.19124.r27606

**Published:** 2020-01-07

**Authors:** Julie Hunt

**Affiliations:** 1 Lincoln Memorial University

This review has been migrated. The reviewer awarded 3 stars out of 5 This article investigates the link between students' dissatisfaction with their Nigerian medical education and their intent to migrate upon graduation for further medical training. The authors do a good job of framing up and discussing the challenges facing the medical education system in Nigeria specifically. I would have been interested to hear how the students' intent to migrate compared to other schools in the developing world--e.g. 33% of the students intend to pursue specialty training internationally; is that in line with other developing nations, or is that high?I would also like to have seen the full survey results split by school. How many students intended to pursue specialty training at the Nigerian medical schools where students were more satisfied with their education? Also, I hesitate to draw conclusions about the opinions offered by parents or house officers, as there were just 3 and 4 of them interviewed, respectively. If the authors wanted to draw conclusions from those participants, they probably should've interviewed a larger number of those participants. However, the responses from the students and staff, with 10 participants respectively, were probably more adequate for drawing conclusions.For transparency, I am an Associate Editor of MedEdPublish. However I have posted this review as a member of the review panel, so this review represents a personal, not institutional, opinion.

Reviewer Expertise:

NA

No decision status is available
